# High-Density Lipoprotein and Prostate Cancer: An Overview

**DOI:** 10.2188/jea.JE20130006

**Published:** 2013-09-05

**Authors:** Kazuhiko Kotani, Yoshitaka Sekine, Shizukiyo Ishikawa, Imoh Z. Ikpot, Kazuhiro Suzuki, Alan T. Remaley

**Affiliations:** 1 Cardiopulmonary Branch, National Heart Lung and Blood Institute, National Institutes of Health, Bethesda, MD, USA; 1 Cardiovascular and Pulmonary Branch, Cardiopulmonary Branch, National Heart Lung and Blood Institute, National Institutes of Health (NIH); 2 Department of Clinical Laboratory Medicine, Jichi Medical University, Shimotsuke, Tochigi, Japan; 2 自治医科大学臨床検査医学; 3 Department of Urology, Gunma University Graduate School of Medicine, Maebashi, Japan; 3 群馬大学医学部泌尿器科学; 4 Division of Community and Family Medicine, Center for Community Medicine, Jichi Medical University, Shimotsuke, Tochigi, Japan; 4 自治医科大学地域医療学

**Keywords:** epidemiology, HDL-cholesterol, apoA-I, prostate tumor

## Abstract

Prostate cancer is a common disease in modern, developed societies and has a high incidence and mortality. High-density lipoprotein cholesterol (HDL-C) has recently received much attention as a possible risk marker of prostate cancer development and prognosis. In the present article, we summarized findings from epidemiologic studies of the association between HDL-C and prostate cancer. Low HDL-C level was found to be a risk and prognostic factor of prostate cancer in several epidemiologic studies, although the overall linkage between HDL and prostate cancer has not been definitively established. The mechanisms for this association remain uncertain; however, limited data from experimental studies imply a possible role of HDL in the pathophysiology of prostate cancer. More epidemiologic research, in combination with experimental studies, is needed in this field.

## Abstract (ja)

今日、前立腺がんの発症あるいはこれによる死亡は高率にみられ、前立腺がんはありふれた疾患の一つと考えられている。高比重リポ蛋白（high-density lipoprotein：HDL）は、最近、前立腺がんの発症や予後に関する指標になる可能性が示唆されるようになり、非常に注目されている。そこで、われわれは血清HDLコレステロール値と前立腺がんの関係を調査した疫学研究を総説した。いくつかの研究は、前立腺がんの発症または予後と低HDLコレステロール血症の有意な関連を見出していたが、他方で複数の研究が関連は乏しいとしていた。すなわち、HDLと前立腺がんの関係には可能性が示唆されるものの、確定的とまでは断言できないのが現状と思われた。両者の関連性についての生物学的機序に関する実験研究は、HDLの前立腺がんへの関与を示唆している。HDLと前立腺がんの関係については、さらに多くの疫学研究の実施が期待されている。

## INTRODUCTION

**Figure 1.**
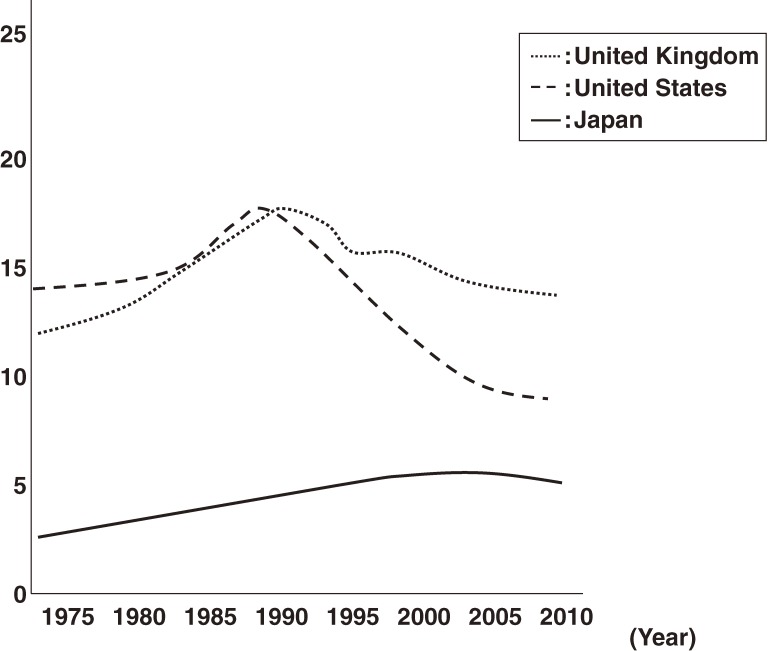
Trends in prostate cancer mortality in the United Kingdom, United States, and Japan; age-standardized rate per 100 000; WHO (www.who.int/gho).

Prostate cancer is common in almost all Western countries.^1^ In the United States, it is the most commonly diagnosed cancer and ranks second in terms of cancer mortality.^1^ As shown in Figure 1, prostate cancer mortality has recently decreased in Western countries.^2^ However, the incidence and mortality of prostate cancer are increasing in Japan.^3^ In addition to age, race, and family history of prostate cancer, the documented risk factors for prostate cancer include environmental and lifestyle-related factors such as obesity, physical inactivity, and a high-fat diet.^4^^,^^5^ The incidence of prostate cancer among Japanese immigrants to the United States is 4 times that of Japanese living in Japan,^6^ suggesting a significant contribution of environmental and lifestyle-related factors to prostate cancer development. One study found that a 45-g increase in total fat consumption per day was significantly associated with prostate cancer development.^7^ The recent increase in fat consumption among Japanese is assumed to be 1 reason why prostate cancer incidence is increasing in Japan.^3^

Several markers, including the prostate-specific antigen (PSA) test, are used to identify patients with prostate cancer. Identification of risk factors and screening tools are important in preventing and treating prostate cancer. Recently, the possibility that high-density lipoprotein cholesterol (HDL-C) and HDL particles are associated with cancer has generated considerable interest.^8^ Lipoproteins, particularly HDL particles, are essential components in the reverse cholesterol transport pathway, the system whereby lipid homeostasis in peripheral tissues is controlled by removal of excess cellular cholesterol by HDL and delivery to the liver for excretion.^8^ Blood levels of HDL-C are affected by chronic low-grade inflammatory diseases such as cancer, but HDL may also have a direct pathophysiologic role in prostate cancer development and/or progression.^8^ Because this is a relatively new field, involving several different disciplines, this research topic has not been previously reviewed. The present review article summarizes current information on the relationship between HDL and prostate cancer, which we hope will stimulate future research in this area.

## EPIDEMIOLOGIC FINDINGS OF ASSOCIATIONS BETWEEN HDL-C AND PROSTATE CANCER

Using a PubMed-based search engine, we selected 13 relevant articles^9^^–^^21^ published up to August 2012. The keywords used in the search were HDL and prostate cancer, and all original reports of epidemiologic studies were eligible for inclusion. Some studies focused on a hyperinsulinemia-related phenotype or some entity of metabolic disorder^12^^,^^14^^,^^18^^,^^19^^,^^21^ but were included if HDL-C level was statistically analyzed as an independent variable and information relevant to prostate cancer was provided. The appropriateness of all articles used in the present review was agreed upon by 2 of the authors. Given the association of HDL-C with study outcomes of interest, the articles are summarized in Table 1 (HDL-C as a risk factor) and Table 2 (HDL-C as a prognostic factor). Two articles that investigated HDL-C as both a risk and prognostic factor of prostate cancer^14^^,^^16^ are included in both Tables 1 and 2.

**Table 1.** Summary of epidemiologic studies of HDL-C as a risk factor in prostate cancer

Author, year	Country	Setting	Age, years (mean or range)	No. of subjects (cancer cases)	Outcomes of interest	Main results	Adjusted covariates	Conclusions	Notes
**Case-control studies**									
Magura L, 2008 (ref. 9)	USA	Hospital	50–74	631 (312)	Cancer: histopathology	ASignificant association between low HDL-C and cancer (OR: 1.57 [95% CI: 1.04–2.36]).	Age, family history of prostate cancer, BMI, type 2 diabetes, smoking, multivitamin use, statin use	**Positive +** Low HDL-C associated with cancer.	
Grosman H, 2010 (ref. 10)	Argentina	Hospital	50–65	150 (50)	Cancer: histopathology	Cancer patients had lower HDL-C (0.88 mmol/L) vs. controls (1.20 mmol/L) and benign prostatic hyperplasia (1.14 mmol/L).	None	**Positive +** Low HDL-C associated with cancer.	Age- and BMI-matched design. HDL-C was inversely correlated with PSA level.
Mittal A, 2011 (ref. 11)	Nepal	Hospital	Cases: 69 (controls: 67)	1200 (600)	Cancer: histopathology	No difference in HDL-C between cases (1.11 mmol/L) and controls (1.11 mmol/L).	None	**No −** HDL-C not associated with cancer.	
**Nested case-control studies**									
Tuohimaa P, 2007 (ref. 12)	Finland	Work-sites	40–58	588 (132)	Cancer: information from registry system	No significant difference in HDL-C between cases (1.28 mmol/L) and controls (1.27 mmol/L). No significant association between low HDL-C (<1.05 mmol/L) and cancer (OR: 0.95 [95% CI: 0.60–1.50]).	Vitamin D	**No −** HDL-C not associated with cancer development.	Age, sampling date, region, and lipid treatment status-matched design. Follow-up period of cohort: 10.8 years. Study focused on the association between metabolic abnormal factors, such as a low HDL-C, and cancer in relation to vitamin D. HDL-C level positively correlated with vitamin D level.
**Cohort studies**									
Ahn J, 2009 (ref. 13)	Finland	General	50–69	29 093 (1586)	Incidence of various cancers, including prostate cancer: medical records with review of histopathology	No significant association between low HDL-C and cancer (HR: 0.89 [95% CI: 0.75–1.06], 5th [>1.43 mmol/L] vs. 1st quintile [<0.94 mmol/L]), and the association was further attenuated after excluding cases from the first 9 years (HR: 0.94 [0.75–1.16]).	Age, intervention, education, systolic blood pressure, BMI, physical activity, smoking duration, number of cigarettes smoked per day, saturated fat intake, total calorie intake, alcohol consumption, serum cholesterol	**No −** HDL-C did not predict cancer development. (Result might be partially affected by reverse causation and preclinical cancer effects)	Follow-up period of cohort: 18 years [median 14.9 years] after a randomized controlled trial with α-tocopherol, β-carotene, or both. The study population was restricted to smokers at study entry. A significant inverse association between HDL-C and some types of cancers (ie, liver and lung cancers) was observed.
Martin RM, 2009 (ref. 14)	Norway	General	≥20	29 364 (687)	Incidence of cancer: information from registry system	No significant association between low HDL-C and cancer (HR: 0.93 [95% CI: 0.76–1.14]; ≥1.5 vs. <1.1 mmol/L).	Age, height, smoking, marital status, education, physical activity, International Prostate Symptom Score	**No −** HDL-C did not predict cancer development.	Follow-up period of cohort: 9.3 years. Low HDL-C was considered as a component of metabolic syndrome in this study. This study also investigated cancer mortality (see Table 2).
Kok DE, 2011 (ref. 15)	Netherland	General	62	2842 (64)	Incidence of cancer: information from registry system	Low HDL-C was significantly positively associated with nonaggressive cancer (HR: 4.28 [95% CI: 1.17–15.67]), but this association disappeared after excluding cases within a 12-month follow-up (HR: 3.03 [95% CI: 0.73–12.51]).	Age, BMI, diabetes history	**No −** HDL-C did not clearly predict cancer development.	Follow-up period of cohort: 79.5 months.
Mondul AM, 2011 (ref. 16)	Finland	General	50–69	29 093 (2041)	Incidence of cancer: medical records or information from registry system	Low HDL-C was suggested to be positively associated with cancer (<40 mg/dL, referent; 40–<60 mg/dL, HR: 0.92 [95% CI: 0.83–1.01]; ≥60 mg/dL, HR: 0.89 [0.77–1.03]).	Age, serum α-tocopherol, family history of prostate cancer, education, urban residence (with additional adjustment for intervention, cigarettes, physical activity, BMI, marital status, total energy, total fat, fruit, vegetable, red meat, alcohol, dietary retinol, vitamin A, vitamin D, calcium)	**No − but borderline** Low HDL-C nonsignificantly but suggestively predicted cancer development.	Follow-up period of cohort: 21 years after a randomized controlled trial with α-tocopherol, β-carotene, or both. The study was an expansion of a prior study by Ahm, 2009 (ref. 13). This study also investigated cancer severity/mortality (see Table 2).
Van Hemelrijck M, 2011 (ref. 17)	Sweden	General	≥35	69 735 (2008)	Incidence of cancer: information from registry system	Low HDL-C was significantly positively associated with cancer (HR: 0.81 [95% CI: 0.70–0.94], the 4th [>1.65 mmol/L] vs. 1st quartiles [<1.13 mmol/L]).	Glucose, triglycerides, fasting status, socioeconomic status	**Positive +** Low HDL-C predicted cancer development.	Follow-up period of cohort: 11.5 years. ApoA-I and HDL-C showed similar trends.

HDL-C: high-density lipoprotein cholesterol, PSA: prostate-specific antigen, BMI: body mass index, OR: odds ratio, HR: hazard ratio.

**Table 2.** Summary of epidemiologic studies of HDL-C as a prognostic factor in prostate cancer

Author, year	Country	Setting	Age, years (mean or range)	No. of subjects (cancer cases)	Outcomes of interest	Main results	Adjusted covariates	Conclusions	Notes
**Cross-sectional studies**									
Hammarsten J, 2004 (ref. 18)	Sweden	Hospital	73	299 (299)	Cancer severity: histopathology	Patients with high-grade cancers had a lower HDL-C (1.10 mmol/L) than those with low-grade cancers (1.28 mmol/L).	BPH growth rate, uric acid, alanine aminotransferase (these were used only in analyzing the subpopulation with PSA < 50 ng/mL)	**Positive +** Patients with more-severe cancer had lower HDL-C.	Low HDL-C was analyzed as a manifestation of hyperinsulinemia in this study.
Prabhat P, 2010 (ref. 19)	India	Hospital	67.5	50 (50)	Cancer severity: histopathology	Patients with high-grade cancers had a lower HDL-C level (0.84 mmol/L) than those with non-high-grade cancers (0.95 mmol/L).	No adjusted factors	**Positive +** Patients with more-severe cancer had lower HDL-C.	This was a small pilot study. Low HDL-C was considered as a metabolic abnormality, as were obesity and hyperinsulinemia.
**Nested case-control studies**									
Jacobs EJ, 2012 (ref. 20)	USA	General	50–79	14 241 (236)	Cancer severity: information from registry system	No significant association between low HDL-C and aggressive cancer (OR: 0.92 [95% CI: 0.54–1.57] in the 4th [≥1.32 mmol/L of HDL-C] vs. 1st quartiles [<0.93 mmol/L] or OR per SD of 0.32 mmol/L: 95% CI: 0.97 [0.82–1.16]).	Physical activity, education, PSA testing history, family history of prostate cancer, heart attack, use of cholesterol-lowering drugs, aspirin use, acetaminophen use, BMI, diabetes.	**No −** HDL-C level was not associated with development of aggressive cancer.	Age- and race-matched design. Follow-up period of cohort: 6–10 years.
**Cohort studies**									
Hammarsten J, 2005 (ref. 21)	Sweden	Hospital	Deaths (cases): 74, survivors (cases): 71	320 (320; deaths: 54)	Mortality of cancer: information of registry and physician network system	Patients who died had a lower HDL-C level (1.18 mmol/L) than those who were alive (1.25 mmol/L).	No adjusted factors	**Positive +** Low HDL-C predicted cancer death.	Follow-up period of cohort: 1233 days. Low HDL-C was analyzed as a manifestation of hyperinsulinemia in this study.
Martin RM, 2009 (ref. 14)	Norway	General	≥20	29 364 (687; deaths: 110)	Cancer severity; mortality of cancer: information from registry system	No significant association was found between a low HDL-C and localized (HR per SD of 0.3 mmol/L: 0.92 [95% CI: 0.80–1.05]) or advanced cancer (HR: 1.08 [0.92–1.25]). No significant association was found between a low HDL-C and the cancer death (HR: 1.03 [0.87–1.23]).	Age, height, smoking, marital status, education, physical activity, International Prostate Symptom Score	**No −** HDL-C level did not predict development of localized/advanced cancer or cancer death.	Follow-up period of cohort: 9.3 years. Low HDL-C was analyzed as a component of the metabolic syndrome in this study. This study also investigated cancer incidence (see Table 1).
Mondul AM, 2011 (ref. 16)	Finland	General	50–69	29 093 (2041)	Cancer severity: medical records or information from registry system	A low HDL-C level was suggested to be positively associated with non-aggressive cancer (<40 mg/dL, referent; 40–<60 mg/dL, HR: 0.88 [95% CI: 0.76–1.02]; ≥60 mg/dL, HR: 0.85 [0.67–1.07]), aggressive cancer (<40 mg/dL, referent; 40–<60 mg/dL, HR: 1.02 [0.83–1.25]; ≥60 mg/dL, HR: 0.89 [0.65–1.22]) and stage ≥3 (<40 mg/dL, referent; 40–<60 mg/dL, HR: 0.87 [0.65–1.17]; ≥60 mg/dL, HR: 0.85 [0.60–1.19]).	Age, serum α-tocopherol, family history of prostate cancer, education, urban residence (an additional adjustment used intervention, cigarettes, physical activity, BMI, marital status, total energy, total fat, fruit, vegetable, red meat, alcohol, dietary retinol, vitamin A, vitamin D, calcium)	**No − but borderline** Low HDL-C nonsignificantly but suggestively predicted cancer development, regardless of severity.	Follow-up period of cohort: 21 years after a randomized controlled trial with α-tocopherol, β-carotene, or both. The study was an expansion of prior study by Ahm, 2009 (ref. 13). This study also investigated cancer incidence (see Table 1).

HDL-C: high-density lipoprotein cholesterol, PSA: prostate-specific antigen, BMI: body mass index, OR: odds ratio, HR: hazard ratio.

### HDL-C as a Risk Factor

Of the 9 studies that investigated the association between prostate cancer and HDL-C as a risk factor, 3 were case-control studies,^9^^–^^11^ 1 was a nested case-control study,^12^ and 5 were prospective cohort studies.^13^^–^^17^ Three studies were conducted in Finland.^12^^,^^13^^,^^16^ All case-control studies were hospital-based.^9^^–^^11^ The nested case-control study investigated a nonspecific population of workers.^12^ All cohort studies investigated general populations.^13^^–^^17^

Overall, the hospital-based studies investigated older patients (age >50 years).^9^^–^^11^ The sample sizes of these hospital-based studies were relatively small (a maximum of 600 patients with prostate cancer), and diagnosis of prostate cancer was based on prostate histopathology.^9^^–^^11^ Most cohort studies of general populations used data from a registry system to obtain information on patients with prostate cancer.^14^^–^^17^ Most cohort studies had large sample sizes (>20 000 subjects), and the study follow-up periods were approximately 10 years or longer.^13^^,^^14^^,^^16^^,^^17^ Statistical adjustment for confounding factors was not uniform and varied greatly between studies.

Three studies found a significant positive relationship between low HDL-C level and the prevalence^9^^,^^10^ and incidence of cancer,^17^ and a borderline positive relationship between low HDL-C level and cancer incidence was observed in 1 study.^16^ In contrast, no relationship was observed between HDL-C and cancer in 5 studies.^11^^–^^15^ In relation to study design, 2 of the 3 case-control studies showed positive relationships.^9^^,^^10^ Among the 5 cohort studies, a positive relationship was seen in 1 study,^17^ and a possible positive relationship was observed in 1 study.^16^ The positive relationship was reported in the cohort study with the largest sample.^17^ In relation to study setting, 2 of the 3 hospital-based studies showed positive relationships,^9^^,^^10^ and, in the 5 studies of general populations, a positive relationship was seen in 1 study,^17^ and a possible positive relationship was seen in another study.^16^

### HDL-C as a Prognostic Factor

Of the 6 studies that investigated the association between prostate cancer and HDL-C as a prognostic factor, 2 were cross-sectional studies,^18^^,^^19^ 1 was a nested case-control study,^20^ and 3 were prospective cohort studies.^14^^,^^16^^,^^20^ Two studies were conducted in Sweden.^18^^,^^21^ All cross-sectional studies were hospital-based.^18^^,^^19^ One nested case-control study investigated a general population.^20^ One cohort study was hospital-based,^21^ and the other cohort studies investigated general populations.^14^^,^^16^

The overall findings from these studies were similar to those of the above-mentioned studies of the association between prostate cancer and HDL-C as a risk factor.^9^^–^^17^ The hospital-based studies investigated patients older than 50 years,^18^^,^^19^^,^^21^ and the sample sizes of these hospital-based studies were relatively small.^18^^,^^19^^,^^21^ Diagnosis of prostate cancer was generally based on prostate histopathology.^18^^,^^19^ The nested case-control study and cohort studies used registry systems to obtain information on patients with prostate cancer.^14^^,^^16^^,^^20^^,^^21^ The sample sizes of cohort studies of general populations were large (>20 000 subjects), and the follow-up period was approximately 10 years or longer.^14^^,^^16^ Adjusted for confounding factors varied considerably among the studies.

A significant positive relationship between low HDL-C levels and cancer severity was reported in 2 studies,^18^^,^^19^ and a possible positive relationship between low HDL-C levels and cancer severity was found in 1 study.^16^ A significant positive relationship between low HDL-C and cancer mortality was also observed in 1 study.^21^ In contrast, 2 studies found no relationship between HDL-C and cancer severity^14^^,^^20^ or mortality.^14^ In relation to study design, 2 cross-sectional studies found positive relationships.^18^^,^^19^ Among the 3 cohort studies, a positive relationship was seen in 1 study^21^ and a possible positive relationship was seen in another study.^16^ In relation to study setting, 3 of the 3 hospital-based studies showed positive relationships,^18^^–^^21^ and a possible positive relationship was seen in 1 of the 3 studies^16^ of general populations.

In sum, the results of the several epidemiologic studies show that blood HDL-C might be a risk or prognostic factor for prostate cancer. The cohort study with the largest sample of a general population, which adjusted for most potential confounding factors, showed a significant association between low HDL-C level and prostate cancer incidence.^17^ However, epidemiologic evidence of a link between HDL-C and prostate cancer is mixed: about half of the studies showed a positive association and the other half showed no association. It is important to note that these studies differed in design, population, and covariate adjustment. In our view, a link between HDL-C and prostate cancer has not yet been definitively established.

Blood HDL-C level is influenced by several lifestyle-related factors, such as obesity and dietary components.^22^ These factors, including obesity and diet (eg, high intakes of dairy protein and fat and low intake of fish), were also reported to be associated with prostate cancer incidence and mortality.^4^^,^^5^^,^^7^^,^^23^ However, not all of the presently reviewed studies adjusted for these factors. The current situation might be resolved in future studies, including cohort studies with larger samples of varied populations, longer follow-up periods, and full consideration of adjusted factors. Studies of selected populations, such as Japanese people with recent changes in diet components and increased incidence of prostate cancer, are warranted. In addition, intervention studies on lifestyle factors and drugs that modify blood HDL-C levels may be undertaken in the future.

## BIOLOGICAL MECHANISMS FOR THE POSSIBLE ASSOCIATIONS BETWEEN HDL AND PROSTATE CANCER

Recent experimental studies can help explain the biological mechanisms for the positive association between HDL and prostate cancer observed in epidemiologic studies. The experimental studies used HDL-related apolipoproteins (ie, apolipoprotein A-I) and their mimetic peptides to generate information on the association between HDL and cancer cells.^24^^–^^26^ One study found that HDL mimetic peptide significantly reduced viability and proliferation of colon cancer cells and that the cell-mediated cancer burden was lower in BALB/c mice.^24^ The same mimetic peptide yielded similar results when tested against ovarian cancer.^25^ These experimental findings of a direct effect of HDL on cancer cells are evidence of a pathophysiologic relationship between HDL-C and prostate cancer, although the relationship between HDL mimetic peptide and prostate cancer requires confirmation.

**Figure 2.**
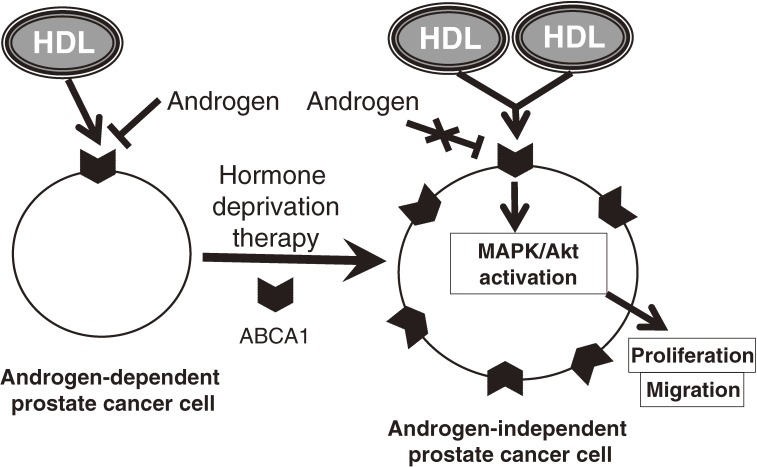
Model of the effect of HDL, via ABCA1, on a prostate cancer cell. HDL: high-density lipoprotein, ABCA1: ATP-binding cassette subfamily A member 1.

Additional points to be considered in interpreting epidemiologic findings are the androgen status and androgen-dependency of the cancer. While increased uptake of HDL in cells resulted in higher rates of cancer cell progression,^27^^–^^30^ patients with prostate cancer who received androgen deprivation therapy had an increase in HDL-C level.^31^ This seemingly paradoxical correlation suggests that consideration of androgen and its related cell characteristics may deepen understanding of the association between HDL-C and prostate cancer. An important concern is if HDL directly affects prostate cancer progression it may do so by inducing androgen-“independent” rather than androgen-“dependent” cell progression of prostate cancer. Androgen-independent prostate cancer cells basically occur after androgen deprivation therapy. We previously described the direct effect of HDL on human prostate cancer cell lines.^32^ We found that HDL induced cell proliferation and migration of androgen-independent prostate cancer cells by a mechanism involving ERK1/2 and Akt, whereas HDL had no such effects on androgen-dependent prostate cancer cells.^32^ With regard to receptors/transporters of HDL, we found that knockdown of ATP-binding cassette subfamily A member 1 (ABCA1)—but not ATP-binding cassette subfamily G member 1 or scavenger receptor class B member I (SR-BI)—inhibited HDL-induced cell proliferation in androgen-independent prostate cancer cells.^32^ Androgens suppress ABCA1 expression in prostate cancer cells,^33^ and ABCA1 is more abundantly expressed in androgen-independent prostate cancer cells than in androgen-dependent cells.^34^ HDL through ABCA1 thus mediates signal transduction, thereby promoting proliferation and migration of androgen-independent prostate cancer cells (Figure 2).

**Figure 3.**
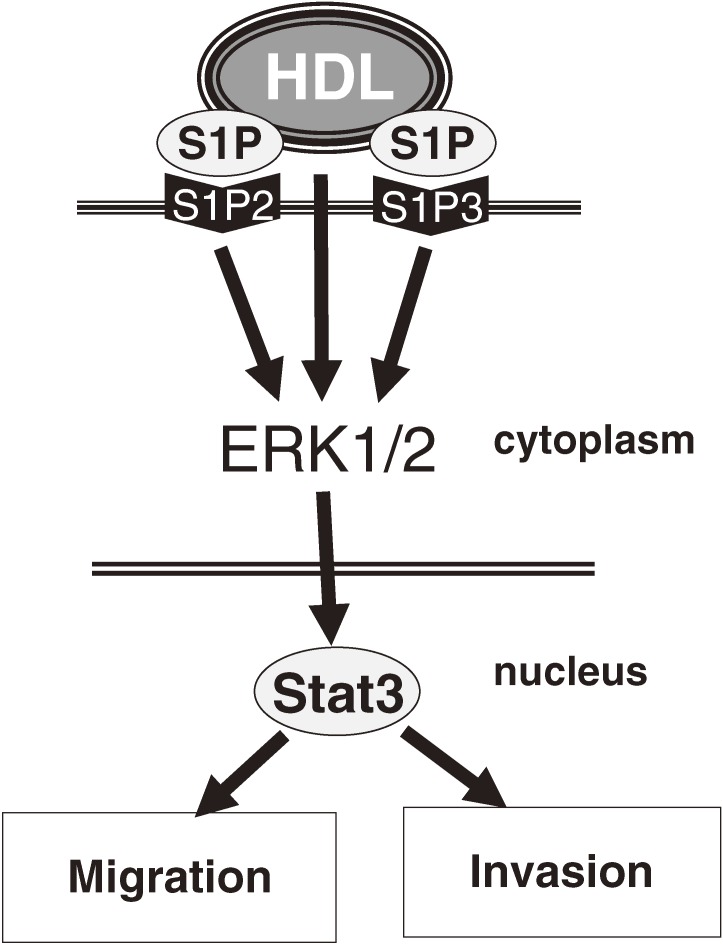
Model of the effect of HDL, via S1P, on a prostate cancer cell. HDL: high-density lipoprotein, S1P: sphingosine-1-phosphate.

We then examined the role of sphingosine-1-phosphate (S1P), a potent bioactive lipid that is partially delivered to cells by HDL.^35^ Both S1P and S1P with reconstituted HDL activated Stat3, which is involved in cell migration, invasion, proliferation, and apoptosis in both normal cells and androgen-independent prostate cancer cells.^35^ The S1P2 and S1P3 receptors were essential in the activation of Stat3 (Figure 3).^35^ Thus, in addition to ABCA1, S1P has a relevant role in HDL-induced cell progression of androgen-independent prostate cancer.

Extant epidemiologic studies of the relationship between HDL-C and prostate cancer did not consider androgen levels or HDL pathway-related molecules, or the relation to the androgen-dependency of cancer. In addition, no epidemiologic study has investigated a specific population with castration-refractory prostate cancer. The interaction between androgen and the HDL pathway may be complex, but a better understanding of this interaction could increase our knowledge of the association between HDL and prostate cancer.

## SUMMARY AND PERSPECTIVE

Although the prevalence of prostate cancer is decreasing in some countries, it is increasing in others, such as Japan, and prostate cancer remains a major cause of cancer mortality in developed countries. Low HDL-C was reported to be a risk and prognostic factor for prostate cancer in several epidemiologic studies. However, current evidence does not conclusively show such an association. More epidemiologic studies, including cohort studies with larger samples of various populations, longer follow-up periods, and full adjustment for covariates, are necessary to confirm this association. Although only a few experimental studies have assessed the impact of HDL on prostate cancer, the findings imply that HDL has a pathophysiologic role in prostate cancer. More epidemiologic studies, in combination with experimental work, are needed in this field.

## ONLINE ONLY MATERIALS

Abstract in Japanese.
