# Supplementary material for: High-Density Lipoprotein and Prostate Cancer: An Overview
Source: J Epidemiol. 2013 Sep 5;23(5):313–9. doi: 10.2188/jea.JE20130006 (PMC3775524; doi:10.2188/jea.JE20130006)
Supplement: Abstract in Japanese. [file je-23-313-s001.pdf]

HDLと前立腺がん：総説

小谷和彦[1、2]、関根芳岳[3]、石川鎮清[4]、Imoh Z. Ikpot[1]、鈴木和浩[3]、
Alan T. Remaley[1]

[1]Cardiovascular and Pulmonary Branch、Cardiopulmonary Branch、National Heart Lung and Blood Institute、National Institutes of Health (NIH)
[2]自治医科大学臨床検査医学
[3]群馬大学医学部泌尿器科学
[4]自治医科大学地域医療学

今日、前立腺がんの発症あるいはこれによる死亡は高率にみられ、前立腺がんはありふれた疾患の一つと考えられている。高比重リポ蛋白（high-density lipoprotein：HDL）は、最近、前立腺がんの発症や予後に関する指標になる可能性が示唆されるようになり、非常に注目されている。そこで、われわれは血清 HDL コレステロール値と前立腺がんの関係を調査した疫学研究を総説した。いくつかの研究は、前立腺がんの発症または予後と低 HDL コレステロール血症の有意な関連を見出していたが、他方で複数の研究が関連は乏しいとしていた。すなわち、HDLと前立腺がんの関係には可能性が示唆されるものの、確定的とまでは断言できないのが現状と思われた。両者の関連性についての生物学的機序に関する実験研究は、HDLの前立腺がんへの関与を示唆している。HDLと前立腺がんの関係については、さらに多くの疫学研究の実施が期待されている。

キーワード：高比重リポ蛋白、HDL、HDLコレステロール、前立腺がん
